# Acute and Subchronic Airway Inflammation after Intratracheal Instillation of Quartz and Titanium Dioxide Agglomerates in Mice

**DOI:** 10.1100/tsw.2011.67

**Published:** 2011-04-05

**Authors:** Martin Roursgaard, Keld A. Jensen, Steen S. Poulsen, Niels-Erik V. Jensen, Lars K. Poulsen, Maria Hammer, Gunnar D. Nielsen, Søren T. Larsen

**Affiliations:** ^1^ National Research Centre for the Working Environment, Copenhagen, Denmark; ^2^ Department of Public Health, Section of Environmental Health, University of Copenhagen, Copenhagen, Denmark; ^3^ Department of Biomedical Research, The Panum Institute, University of Copenhagen, Copenhagen, Denmark; ^4^ Laboratory of Medical Allergology, Allergy Clinic, Copenhagen National University Hospital, Gentofte, Denmark

**Keywords:** nanoparticles, quartz, TiO_2_, inflammation, mice, dose response, intratracheal instillation

## Abstract

This study investigated the acute and subchronic inflammatory effects of micrometer-size (micro-size) and nanometer-size (nano-size) particles after intratracheal (i.t.) installation in mice. The role of the type of compound, polymorphism, and size of the particles was investigated. Studied compounds were the two micro-size reference quartzes, SRM1878a and DQ12, a micro- and nano-size rutile titanium dioxide (TiO_2_), a nano-size anatase, and an amorphous TiO_2_. Particles were administered by a single i.t. instillation in mice at a fixed dose of 5, 50, and 500 μg, respectively. Inflammation was evaluated from the bronchoalveolar lavage fluid (BALF) content of inflammatory cells, the cytokines tumor necrosis factor alpha (TNF-α) and interleukin 6 (IL-6), as well as from lung histology. Evaluations were at 24 h (acute effects) and 3 months (subchronic effects) after instillations. Both types of quartz induced a dose-dependent acute increase of neutrophils, IL-6, and total protein in BALF. Limited subchronic inflammation was observed. All types of TiO_2_ induced a dose-dependent acute increase of neutrophils in BALF. In the acute phase, micro- and nano-size rutile and nano-size amorphous TiO_2_ induced elevated levels of IL-6 and total protein in BALF at the highest dose. At the nano-size rutile and amorphous TiO_2_, subchronic lung inflammation was apparent from a dose-dependent increase in BALF macrophages. Histology showed little inflammation overall. The two types of quartz showed virtually similar inflammatory effects. Nearly similar effects were observed for two sizes of rutile TiO_2_. Differences were seen between the different polymorphs of nano-size TiO_2_, with rutile being the most inflammogenic and amorphous being the most potent in regard to acute tissue damage.

### INTRODUCTION

Inorganic particles can cause severe lung diseases (such as pneumoconiosis and chronic obstructive pulmonary disease [COPD]) and cancer, and can promote the development of allergies[1,2,3,4,5,6,7]. Currently, several new types of particulate chemicals have appeared, including a wide range of engineered nanoparticles. These nanoparticles may be more toxic than coarser particles due to their smaller size and increased specific surface area[8,9,10], but this need not always be the case[11,12,13,14,15].

Due to limitations of *in vitro* methods, animal studies are still necessary for the hazard evaluation and screening of new particles[16,17,18,19,20,21]. The gold standard for pulmonary toxicological evaluation of new substances is long-term inhalation studies, which best mimic real-life exposure conditions. However, as inhalation is a laborious and complicated method, alternative exposure methods, such as intranasal and intratracheal (i.t.) instillation, are often used to mimic inhalation exposure[22,23,24,25,26,27].

In this study, we chose to examine lung inflammation as the end point because inflammation is a key reaction in many particle-induced lung diseases. Quartz was selected as a benchmark material as it is known to induce pulmonary inflammation[28,29,30] and is often used as a positive control in studies of particle-induced inflammation[28,29,31,32,33]. In this study, the NIST SRM1878a and DQ12 quartz materials were used for evaluation and comparison of the inflammatory potencies of different micro- to nano-size titanium dioxide ($TiO_2$) polymorphs. SRM1878a and DQ12 are often-used benchmark materials in other studies and their use enables comparison with other studies using either of these materials. Therefore, it was also a focus of this study to evaluate the inflammogenicity of these two reference materials. To our knowledge, this is the first attempt to compare their inflammatory effects. The other investigated particles for lung inflammation were two rutile $TiO_2$ particles with different sizes, as well as anatase and amorphous $TiO_2$ in the nano size.

$TiO_2$ particles are often used as negative control particles[34,35,36] as they are poorly soluble and often considered to be toxicologically low-potency compounds. However, studies have shown that $TiO_2$ in the nano-size range may possess a higher inflammatory effect than $TiO_2$ in the micro-size range. This questions whether $TiO_2$ is suitable as a low toxic negative control in general[37,38,39,40,41]. In this study, quartz and $TiO_2$ particles were selected in order to cover the range from high to low potency of particles in regard to lung inflammation.

For the analysis, a single i.t. instillation in mice was used. This i.t. instillation was chosen due to simplicity because a well-defined dose can easily be delivered to the lungs and because i.t. instillation only requires a small amount of particles. The metric chosen was mass of particles as present regulations are based on mass of airborne particles. Increasing evidence suggests that surface area, number of particles, or hydrodynamic size of nanoparticles may serve as better descriptors of inflammatory effects[38,42,43]; why their importance was also evaluated.

The inflammatory effects of the particles were determined 24 h after instillation (acute phase) and after 3 months (subchronic phase). Inflammation was determined from the bronchoalveolar lavage fluid (BALF) content of inflammatory cells, tumor necrosis factor alpha (TNF-α), interleukin 6 (IL-6), total protein, and from lung histology. The selected time points and end points were substantiated in a previous study[14].

This study compares the inflammatory effects of the two types of quartz, SRM1878a and DQ12, as well as effects of different particle sizes and polymorphs of $TiO_2$ from biochemical and histological end points. The nanoparticulate $TiO_2$ materials were produced by wet-chemical processes, whereas the micro-size particles were produced by flame-spray synthesis.

## METHODS

### Particles

SRM1878a was obtained from the National Institute of Standards and Technology (NIST) (Washington, D.C., U.S.A.) and consisted of respirable Brazilian α-quartz (93.7 ± 0.2%) with a median particle size of 1.6 µm, measured by powder laser scattering by NIST. The other quartz material was DQ12 (IUF batch 7), which was kindly supplied by Professor Roel Schins (Heinrich-Heine Universität, Düsseldorf, Germany). Micro-size rutile $TiO_2$ was from Sigma-Aldrich. The particle size was <5 µm and the purity ≥99.9% rutile according to the supplier.

All nano-size $TiO_2$ particles were kindly supplied by Professor A. Navrotsky (University of California at Davis, California, U.S.A.); the synthesis of the particles is summarized below and further described in Ranade et al.[44]. The crystallite sizes determined by Rietveld refinement of powder X-ray difractograms and BET (Brunauer-Emmet-Teller) analyses (Table 1) confirms that particles termed “nano” may be classified as nanoparticles according to ISO (2010) and nanomaterials SCENIHR (2010).

**TABLE 1**
**Summary of Selected Sample Characteristics**

| | SRM1878a | DQ12 | $TiO_2$MR | $TiO_2$NR | $TiO_2$NA | $TiO_2$NAM |
|---|---|---|---|---|---|---|
| Phases (wt%) | | | | | | |
| Am $TiO_2$ | 6.3[a] | 13[a] | | | | |
| α-Quartz | 93.7[a] | 87[a] | | | | |
| Am $TiO_2$ | | | — | — | — | 100[c] |
| Rutile | | | 99.9[a] | 100[c] | — | — |
| Anatase | | | — | — | 100 [c] | — |
| Average XRD size (nm) | 155.2[b] | | ND | 8.0[c] | 10.4[c] | NA |
| Average particle size (µm) | 1.6[a] | 1.2 | <5[a] | ND | ND | ND |
| Peak size, $d_H$; saline solution (µm) (volume peak size / width / %[d]) | 1.80 / 0.29 / 91.2%<br>5.56 / 0.59 / 8.8% | 1.64 / 0.36 / 100% | 1.17 / 0.29 / 100% | 5.37 / 0.69 / 25.1%<br>2.24 / 0.66 / 74.9% | 5.59 / 0.58 / 7%<br>1.12 / 0.26 / 93% | 5.45 / 0.65 / 19.3%<br>2.13 / 0.83 / 80.7% |
| Peak size, $d_H$; 0.8 µm filtered (nm) (volume peak size / width / %[d]) | 459 / 174 / 100% | 370 / 84 / 100% | 861 / 244 / 85.5%<br>300 / 67 / 14.6% | 745 / 265 / 100% | NA | 5150 / 777 / 1.9%<br>719 / 401 / 1.4%<br>15 / 5 / 96.9% |
| Hydrodynamic surface area[e] ($m^2$/g) | 1.06 | 1.38 | 1.20 | 0.47 | 1.08 | 0.70 |
| BET surface area ($m^2$/g) | 2.7 | 11.49 | 2.6 | 73[c] | 150[c] | 433[c] |
| Spherical equivalent BET size (nm) | 839 | 197 | 543 | 19 | 10 | 4 |

Am: amorphous; $d_H$: hydrodynamic diameter; NA: not applicable; ND: not determined; $TiO_2$MR: micro-size rutile $TiO_2$; $TiO_2$NA: nano-size anatase $TiO_2$; $TiO_2$NAM: nano-size amorphous $TiO_2$; $TiO_2$NR: nano-size rutile $TiO_2$; XRD: X-ray diffraction.

[a] Details provided from supplier.

[b] Measured by Rietveld analysis of data from powder XRD.

[c] Data from Ranade et al.[44].

[d] Volume percent of the fraction within the specific peak.

[e] A theoretical value, cf. text for further explanation.

**$TiO_2$NAM** was synthesized by mixing titanium ethoxide with ethanol and stirring the solution for 2 h at 273°K. Then, 1.6 mol of water with four drops of acetic acid was added to the solution and aged for 0.5 h. Ranade et al.[44] reported this material to be amorphous in X-ray diffraction (XRD) analysis. In addition to $TiO_2$, the analyzed sample contained minor adsorbed water (0.8 wt%) and carbon (0.6 wt%). The specific surface area, which was calculated using BET data (cf. Table 1), was 433 $m^2$/g.

**$TiO_2$NA** was synthesized by adding water to a mixture between $(NH_4)_2Ti(OH)_2(C_3H_5O_3)_2$ and $CH_3(CH_2)_{15}N(Cl)(CH_3)_3$ under continuous stirring at room temperature until the point of irreversible precipitation. After stirring overnight, the solution was transferred to a sealed stirred Teflon reactor and heated to 343°K for 24 h, followed by 48 h at 373°K. The precipitate was subsequently thoroughly washed with water and calcined for 2 h at 623°K to remove residual chemicals. The sample contained 0.6 wt% adsorbed water and 0.14 wt% carbon. The reported average crystallite size was 10.4 nm (XRD, cf. Table 1) and the specific surface area was 150 $m^2$/g.

**$TiO_2$NR** was produced by the sol-gel method. Precipitates were formed by mixing solutions of $TiCl_4$ and $NH_4OH$, slowly increasing the pH from 0.1 to 2 during stirring for 48 h at 300°K. The precipitate was washed with isopropyl alcohol and dried at room temperature to obtain rutile nanoparticles. The sample contained 6.5 wt% adsorbed water and 0.1 wt% carbon. The reported average crystallite size was 8 nm and the specific surface area was 73 $m^2$/g.

### Particle Size in Instillation Suspensions

Before i.t. instillation in mice, each sample of particles was suspended in pyrogen-free saline (1.25 mg/ml) and treated in an ultrasound bath (47 kHz) for 10 min. The particle size distributions in the instillation media were subsequently documented using dynamic light scatter (DLS) analysis.

### DLS Analysis

The hydrodynamic particle number size distribution in saline suspensions was analyzed using a DLS Zetasizer Nano ZS (Malvern Instruments, U.K.) equipped with a 4-mW, 633-nm He-Ne Class I Laser. Ideally, the DLS system can determine hydrodynamic diameters of particles between 0.6 nm and 6 µm, depending on material type and media. Analyses were performed on unfiltered as well as on 0.8-µm-filtered (cellulose acetate; DISMIC®-25CS; Toyo Roshi Kaisha, Japan) suspensions to determine the presence of smaller particles, which may not be observed in mixtures with coarse particles due to high backscatter intensities from the coarse particles. Analyses were conducted on both high-concentration instillation solutions and on diluted samples to evaluate agglomeration and viscosity effects in the high-concentrated suspensions. Samples were measured in 120-µl disposable polystyrene cuvettes and size distributions were obtained using dispersion technology software (DTS) vs. 5.0 (Malvern Instruments); the number distributions were calculated assuming the viscosity (0.6864 cP) and the refractive index ($R_i$) of water ($R_i$ = 1.330) for the medium. The $R_i$ and the absorption indices ($R_s$) were used for $\alpha$-quartz ($R_i$ = 1.544; $R_s$ = 0.200), rutile ($R_i$ = 2.903; $R_s$ = 0.100), anatase ($R_i$ = 2.490; $R_s$ = 0.100), and amorphous $TiO_2$ ($R_i$ = 2.490; $R_s$ = 0.100). Data quality was ensured by evaluating the intensity correllelograms, cumulant fits, and the distribution fits of the laser scattering intensity data.

### Transmission Electron Microscopy

Particle size, morphology, and *in situ* elemental compositions were obtained using a JEOL 1200EX II scanning transmission electron microscope (STEM) equipped with an energy dispersive spectrometer (EDS). The STEM was operated at an accelerating voltage of 80–100 kV, and imaging was made using Oxford Link software. TEM samples were prepared by adding droplets of particle suspensions to holey carbon-coated copper grids placed on filter paper for quick absorption of the liquid. Samples were allowed to dry for a few minutes in air on the filter paper, covered by a glass lid, and then transferred to individual storage in polymer-embedding capsules until analysis.

### Animals

Female BALB/cJ mice, aged 7–8 weeks (18.5–21.3 g) (Taconic M&B, Denmark), were housed in polypropylene cages (380 × 220 × 150 mm) with pinewood sawdust bedding (Lignocel® S8, Brogaarden,

Denmark). Each cage contained eight mice and was furnished with bedding materials, gnaw sticks, and cardboard tubes. The photoperiod was from 6 a.m. to 6 p.m., and the temperature and mean relative humidity in the room were 19–22˚C and 43 ± 8% (S.D.), respectively. Cages were cleaned twice weekly. Food (Altromin no. 1324, Altromin, Germany) and tap water were available *ad libitum*. Treatment of the animals followed procedures approved by the Animal Experiment Inspectorate, Denmark.

### Selection of Dose

In previously conducted studies in mice, the i.t. administration doses varied from 100 to 7000 µg/mouse. To investigate the low-dose range, we studied the effect of 50 µg/mouse, which induced airway inflammation[14]. In this study, we further investigate the low-dose effects from 5 and 50 µg/mouse by i.t. instillation. To establish dose-response relationship, we also included the 500 µg/mouse dose.

### Selection of Time Points

From published studies, time points can be divided into two groups: ≤7 days (acute phase) and >7 days postinstillation, but no detailed trend in inflammation kinetics was retrieved. Therefore, we previously performed a detailed study in the period 1–48 h postinstillation to investigate the development of biological response in the acute phase[14]. Inflammatory parameters often were maximum about 1 day after an instillation; why this time point was selected for the study of acute effects. Studies where the observation period was >7 days used time points in the interval from 2 weeks to 15 months; mostly the observation period lasted several months. Therefore, in this study, we selected 3 months as the observation period for the screening of subchronic effects as previously proposed[14].

### Selection of End Points in BALF

In our previous mouse study, we investigated lactate dehydrogenase, protein, and inflammatory cell levels, as well as the level of several cytokines. It was concluded that for screening of acute effects of particles, BALF levels of inflammatory cells and either TNF-α or IL-1β was sufficient. In this study, we selected TNF-α as it is involved in several lung diseases, e.g., COPD, asthma, and chronic bronchitis[45,46]. Additionally, we included IL-6 as another inflammatory marker as it was elevated in our previous study[14]. We limited our study to the biologically active cytokines.

### Exposure and BALF Parameters

The different doses of particles (0, 5, 50, and 500 µg/mouse) were administered in a 40-µl vehicle by i.t. instillation in the mice; each group comprised eight animals. For instillation, mice were orotracheally intubated with a flexible polyethylene catheter under deep Hypnorm®/Dormicum® (Jannsen Pharma/Roche) sedation (for details see Jacobsen et al.[22]). At selected time points, 24 h (acute) and 3 months (subchronic) after instillation, mice were euthanized under Hypnorm®/Dormicum® anesthesia and BALF was collected as previously described[47]. Briefly, 200 BALF cells were counted and differentiated into macrophages, neutrophils, lymphocytes, eosinophils, and epithelial cells. TNF-α (limit of detection [LOD] 5.1 pg/ml) and IL-6 (LOD 1.6 pg/ml) in the BALF were determined by ELISA (R&D Systems, U.S.A.) according to the manufacturer's instructions. Total protein in BALF was determined using the BCA Protein Assay Kit (Pierce, U.S.A.) according to the manufacturer's instructions.

### Histology

After collection of BALF, the thorax of the mice was opened in the midline, letting the lungs collapse, and a polyethylene tube was introduced into the trachea. The tube was connected to a syringe containing 4% buffered paraformaldehyde and the lungs were inflated *in situ* with the fixative to their normal size. After 5 min, the lungs were removed *in toto* and further fixated for at least 24 h. Tissues were embedded in paraffin in a standardized way (a horizontal cut through the hilum regions) and, subsequently, 7-µm thick sections were cut on a microtome and stained with hematoxylin/periodic acid Schiff (PAS). The degree of inflammation and morphological changes in the lungs were evaluated blindly by conventional bright-field microscopy by two experienced researchers and revaluated in case of discrepancy as described previously. The presence of particles was also studied by dark-field and polarization microscopy, and scored blinded on a scale from 1 to 5. The degree of inflammation and the amount of particles in the lungs were evaluated blindly by two independent researchers. To study lung fibrosis, sections were stained by van Gieson (stains collagen bright red), and photomicrographs representing an area around a bronchiole and an area with only alveoli were analyzed by means of Image Pro® Plus version 6.0 (MediaCybernetics, U.S.A.).

As the instilled material and inflammatory cells in the lumen were partly removed by the lung lavage, this indicates that intraluminal findings should be considered to be qualitative. In contrast, mesenchymal changes, including accumulation of particles, inflammatory cells, edema, and collagen deposition, can be considered independent of the lung lavage. Also, as lung lavage was performed similarly in the acute and the subchronic phase, findings can be compared across these time points.

### Statistics

Exposure groups were compared pair-wise to the control group by the Mann-Whitney's U-test. The Bonferroni correction was used to obtain an overall significance level of 0.05, 0.01, and 0.001, which are indicated by *, **, and ***, respectively. Since three groups were tested against the corresponding control group, this corresponds to a *p* value of 0.0167, 0.0033, and 0.00033, respectively, in each pair-wise comparison. Where all values in one of the groups were zero, Fisher's Exact Test was used in the statistical comparison. For simplicity, we used percentile statistics for presentation of data[48]. The central finding of results was characterized from the median ($50^{th}$ percentile) and the variance from the $75^{th}$ percentile as also used in Box plots.

### RESULTS

### Particle Characterization

- **Quartz (SRM1878a) —** DLS analysis showed that the stock saline suspension consisted of micro-size particles with a dominant mode around 1.8 and a small mode at 5.6 µm (Fig. 1A and Table 1). Coarser particles were also indicated from the correlation function analysis and a visible rapid sedimentation in the sample. Due to low particle counts, suitable DLS size distribution spectra could not be obtained on the suspension filtered through a 0.8-µm filter, but the presence of 200- to 600-nm-size particles was indicated from light-scattering intensity data (data not shown). STEM and SEM analysis showed the presence of up to approximately 20-µm-size angular quartz grains. Overall, the SRM1878a sample was dominated by coarse particles. The size distribution was wide and ranged far down into the nano-size region (Fig. 2A–C) confirming the vague signal from the DLS analysis of the 0.8-µm-filtered suspension. The smaller particles were often found in agglomerates consisting of a relatively low number of particles (Fig. 2C).

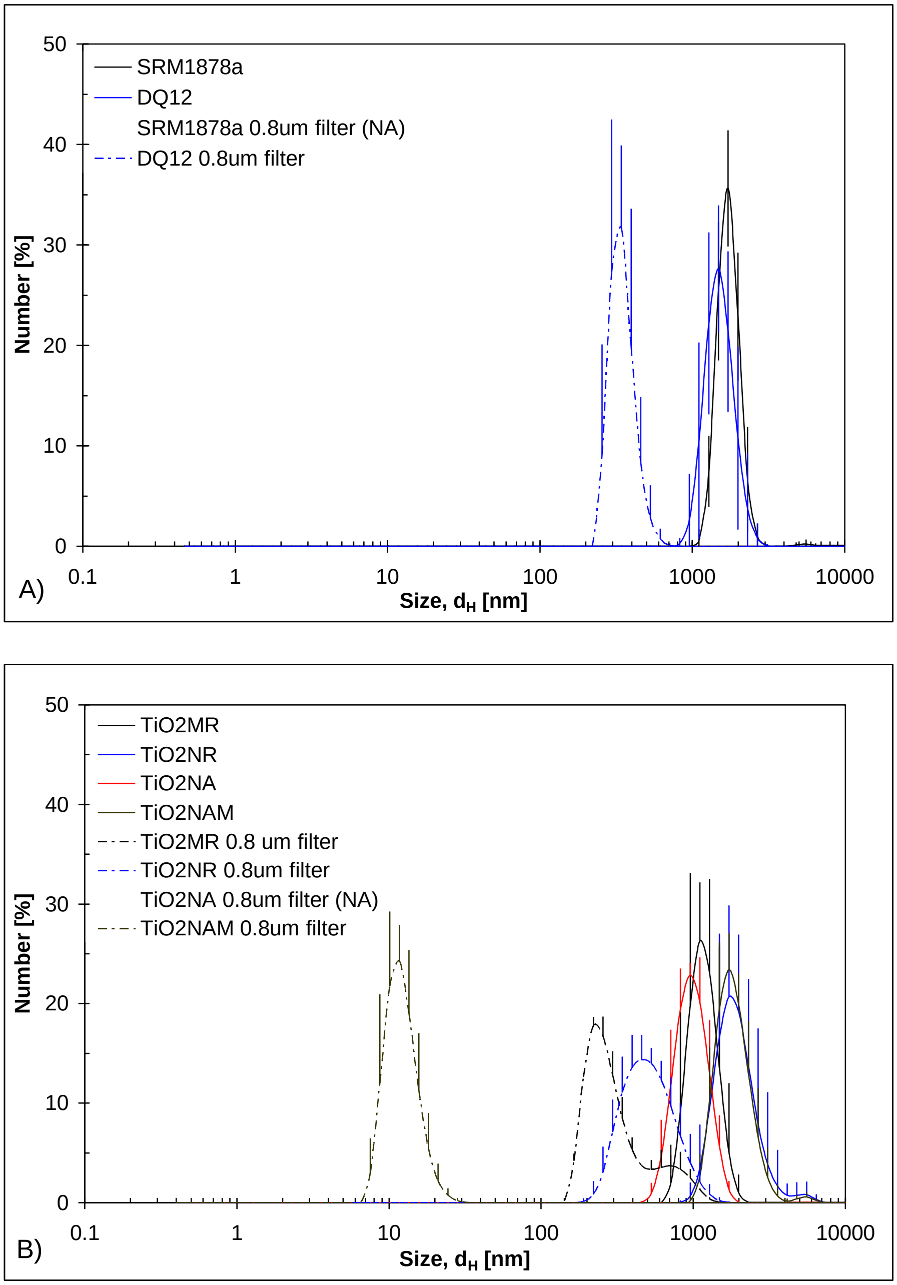

**FIGURE 1.** DLS spectra of the particles in saline. DLS spectra showing the hydrodynamic particle number size distributions ($d_H$) of the saline suspensions used for i.t. instillation. (A) DLS spectra are shown for the stock SRM1878a and DQ12, as well as the 0.8-µm-filtered DQ12 suspensions; it was not possible to obtain a suitable spectrum from the 0.8-µm-filtered SRM1878a suspensions due to a low number of particles. (B) DLS spectra are shown for the stock $TiO_2$ suspensions and 0.8-µm-filtered samples. In (A) and (B), the presence of coarse particles outside the upper detection limit is indicated by an arbitrary x-axis error bar at the coarsest valid size data for SRM1878a and $TiO_2$MR.

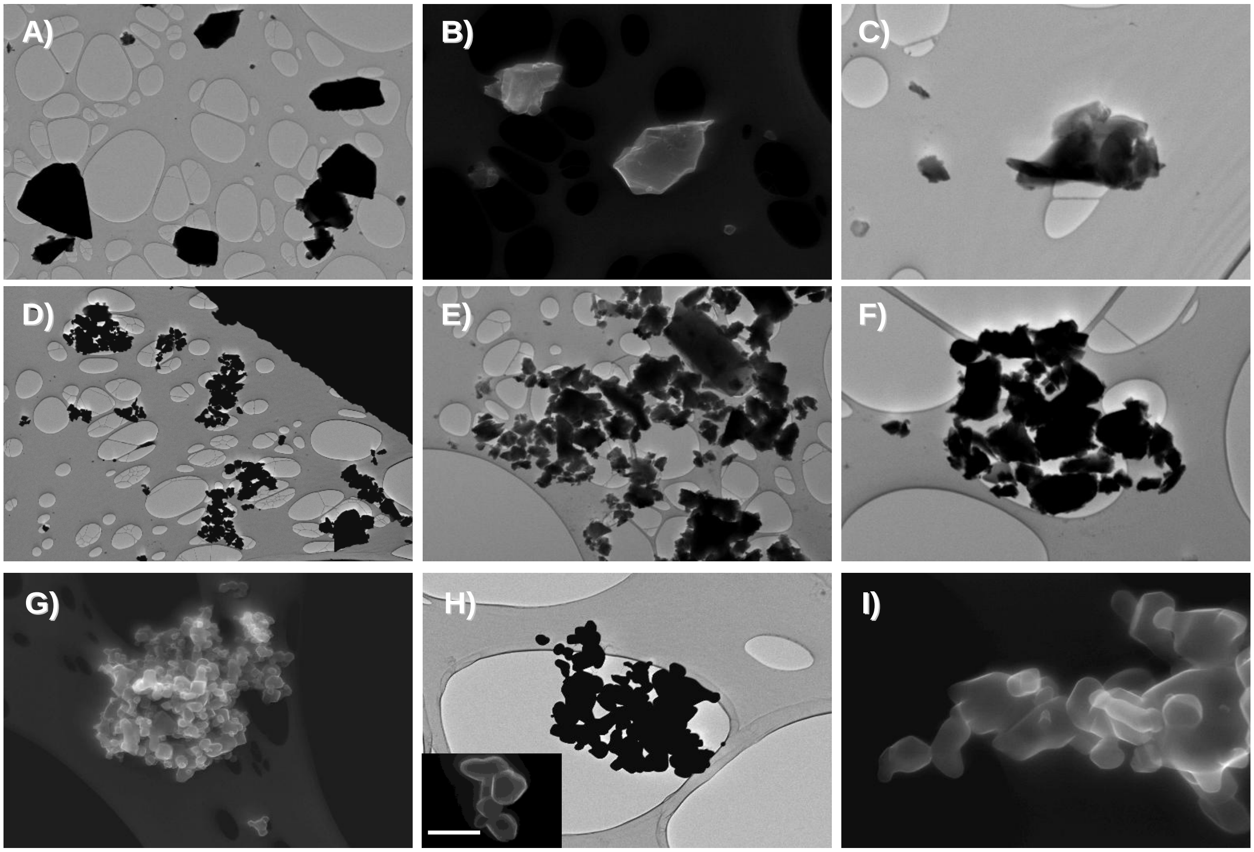

**A–I (figure continues next page)**

**FIGURE 2.** SEM and STEM images of particles from stock solutions prepared in the same manner as those used for i.t. instillations. The scale bar for images in each column is shown in last row (P–R), except where otherwise indicated. (A) STEM image of SRM1878a showing large angular quartz particles and smaller agglomerates. In general, the particles can be seen as dark angular agglomerates or single particles, as opposed to the ellipsoid grid holes. (B) SEM image showing the angular morphology of SRM1878a. (C) STEM image showing the presence of smaller agglomerates/aggregates of quartz in SRM1878a. (D) STEM image of DQ12 showing submicro- to micro-size agglomerates and the individual quartz particles. (E) STEM image showing the open structure of quartz agglomerates in DQ12 as well as the angular morphology of the individual particles. (F) STEM image showing the presence and morphology of smaller particles (<0.5 μm) in DQ12. (G) SEM image showing an example of the partially open aggregate structure of a large aggregate in $TiO_2$MR. (H) STEM image showing an open aggregate of euhedral and a few subhedral rutile particles in $TiO_2$MR. The insert shows a close-up of a small aggregate fused at crystal faces (scale bar 200 nm). (I) SEM image showing euhedral crystals and sub- to anhedral particles fused together in a coarse aggregate in $TiO_2$MR. (J) SEM image showing a general overview of micro- and submicro-size rutile aggregates and agglomerates of $TiO_2$NR. (K) SEM image showing an apparently dense texture of an aggregate/agglomerate cluster in $TiO_2$NR. (L) SEM-image of submicro-size agglomerates, aggregates, and individual rutile crystals in $TiO_2$NR. The insert shows the platy or lathlike shape of the individual rutile nanocrystallites (scale bar 200 nm). (M) SEM image showing an agglomerate consisting of several angular and subangular anatase particles in $TiO_2$NA. (N) SEM image showing small micro- to submicro-size agglomerates/aggregates of individual angular anatase particles in $TiO_2$NA. (O) SEM image showing the rough and highly irregular surfaces of the anatase particles in $TiO_2$NA. (P) SEM image of agglomerated particles in $TiO_2$NAM. (Q) SEM image showing angular to subangular single particles in the nano-size range to a >2-μm aggregate of particles in $TiO_2$NAM. (R) SEM image showing the rough surface of $TiO_2$ particles in $TiO_2$NAM.

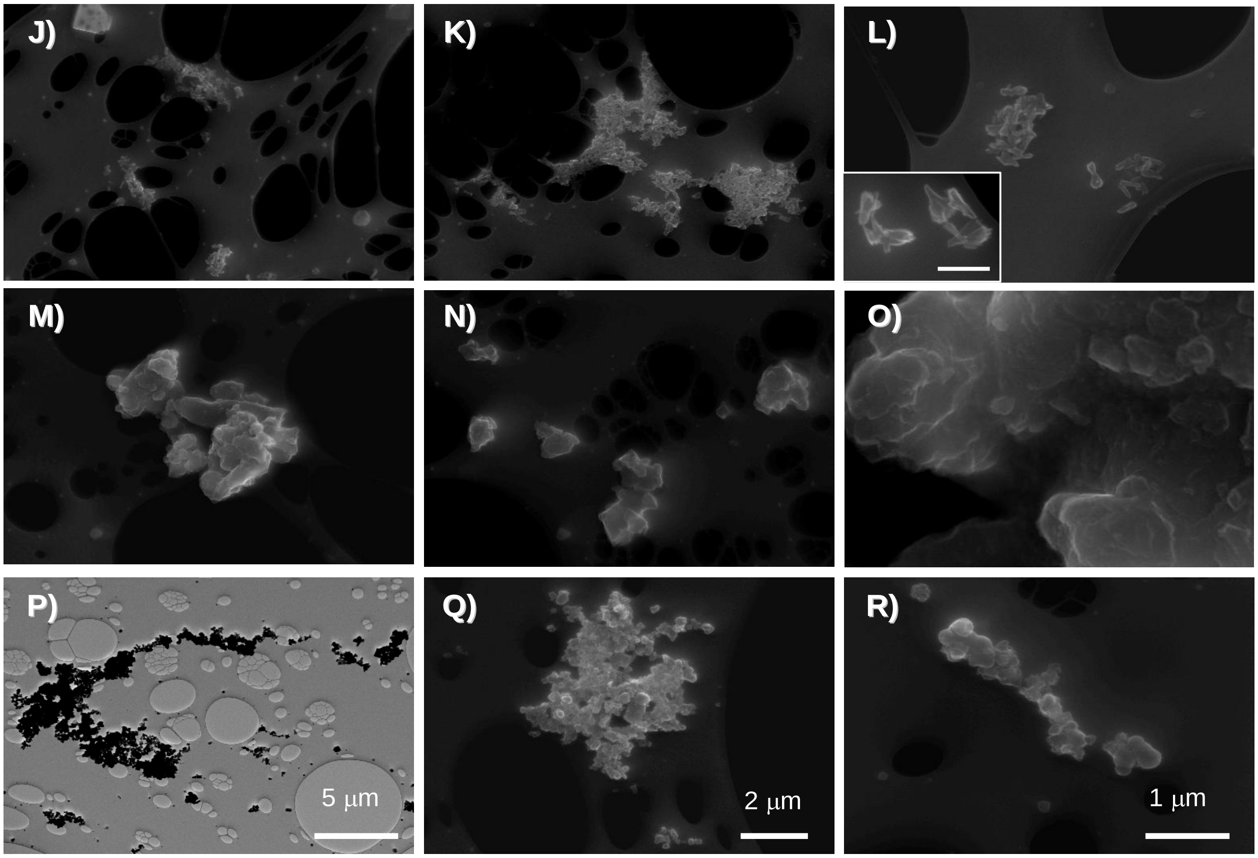

**FIGURE 2J–R**

- **Quartz (DQ12) —** The saline DQ12 suspensions were dominated by micro-size particles with a major mode around 1.6 µm and a very weak mode at approximately 3 µm (Fig. 1A and Table 1). The suspension was slightly affected by sedimentation during the DLS analysis, which suggests that the suspension was unstable. Filtration through a 0.8-µm filter showed a distinct size mode with a peak around 370 nm. SEM and STEM analysis of the saline suspension showed that the individual particles in DQ12 consisted of angular and up to approximately 2-µm particles. However, the typical particle size was much smaller than in SRM1878a (Fig. 2D–F). The particles mostly occurred as loose micro-size agglomerates, but the presence of smaller particles was observed (Fig. 2 D–F) in accordance with the DLS analysis. The smallest particles were <100 nm.
- **Micro-size rutile $TiO_2$ ($TiO_2$MR) —** The DLS analysis was affected by sedimentation in the saline suspension, suggesting the presence of large micro-size particles in the suspension. However, after a short sedimentation period, a metastable suspension with a size mode at approximately 1.2 µm was reached (Fig. 1B and Table 1). The DLS data from the 0.8-µm-filtered sample were of limited reliability due to a low particle count, but two accepted size spectra suggested a broad multimodal size distribution ranging from approximately 100 nm and up into the micro-size range with size mode peaks at 300 and 860 nm. SEM and STEM analysis showed that the $TiO_2$MR particles mainly consisted of aggregated rutile crystals (Fig. 2G–I). The individual particles were dominated by euhedral crystals, but sub- to anhedral particles and "frozen droplets" of $TiO_2$ were also observed, suggesting that there may be a minor fraction of amorphous material in the sample. The aggregate structure was generally relatively open with the individual crystals fused along crystal faces and point contacts (Fig. 2H–I). In accordance with the DLS analysis, the smallest observed primary crystals and aggregates were typically around 100–300 nm (Fig. 2H–I).

- **Nano-size rutile $TiO_2$ ($TiO_2$NR) —** The DLS analysis showed a bimodal size distribution with a size mode around 2 and 5 μm (Fig. 1B and Table 1). Smaller particles with a modal size peak around 460 nm were observed after passing the suspension through a 0.8-μm filter (Fig. 1B). Both suspensions were stable over the measurements, except from some fluctuation for the coarse (5 μm) size mode. SEM and STEM analysis of the particle suspension dried on TEM grids showed the presence of numerous up to several micro-size agglomerates and/or aggregates, as well as minor amounts of individual particles (Fig. 2J–L). The individual rutile particles were typically 20–50 nm wide and 100–200 nm long with a subeuhedral to euhedral tabular morphology (Fig. 2L). The individual particles may consist of flaky crystallites (insert in Fig. 2L), which can explain the small crystallite size (8 nm, Table 1) determined for this sample. The lack of small particles (≤100 nm) in the DLS analysis suggests that the rutile particles were found as agglomerates in saline medium.
- **Nano-size anatase $TiO_2$ ($TiO_2$NA) —** The DLS size distribution showed a peak in the number size distribution at 1.1 μm and a mode at approximately 5.6 μm (Fig. 1B and Table 1). The suspensions were subjected to limited sedimentation during analysis. Filtering through the 0.8-μm filter did not give reliable results due to low counts. However, small nanoparticles with a peak size at approximately 13 nm might be suggested from the laser intensity charts (data not shown). SEM and STEM analysis showed that the anatase occurred in large angular aggregates of up to several micro size and agglomerates (Fig. 2M–O). In contrast to nano-size rutile $TiO_2$, euhedral crystals were not observed in this sample (Fig. 2N). Morphological analysis showed that the particle surfaces were very irregular and rough (Fig. 2O), which in part may explain the high specific surface area determined for this sample (Table 1). Single nano-size particles were not observed. However, nano-size particles detected by DLS showed that small particles were present and nanoparticles were indicated from the BET data (Table 1).
- **Nano-size amorphous $TiO_2$ ($TiO_2$NAM) —** The DLS size distribution of the saline suspension was bimodal with size modes around 2.1 and 5.5 μm, and was almost identical to the suspension with nano-size rutile $TiO_2$ (Fig. 1B). Filtration through the 0.8-μm filter showed the presence of a nanoparticle mode with a peak at ~15 nm as well as small peaks at 720 nm and 5.1 μm (Table 1), indicating further agglomeration in the filtered suspension. SEM and STEM analysis showed that the sample consisted of coarse micro-size to very fine aggregates and agglomerates of mostly irregular anhedral and spherical to semi-spherical $TiO_2$ particles (Fig. 2P–R). In accordance with the nanoparticle mode found in the DLS analysis of filtered suspensions, small agglomerates/aggregates and free individual particles were also observed. The smallest particles were far below 100 nm in size.

### General Description of Histopathological Findings

In general, the inflammatory changes were modest, and were observed as focal peribronchiolar infiltration and inflammation in some areas in the alveolar lumina and in the septa (Fig. 3 and Table 2); particles could easily be identified both by dark-field and polarization microscopy. Particles were taken up by macrophages, but were also present in the connective tissue of the septa. Focal areas with accumulation of macrophages loaded with particles often showed inflammatory changes as well. In the larger bronchi, there might be goblet cell metaplasia (data not shown). When the amount of collagen, stained by means of van Gieson, was quantified and used in order to detect signs of fibrosis, we did not find significant differences between any of the groups, which is in accordance with the only slight interstitial inflammatory changes observed.

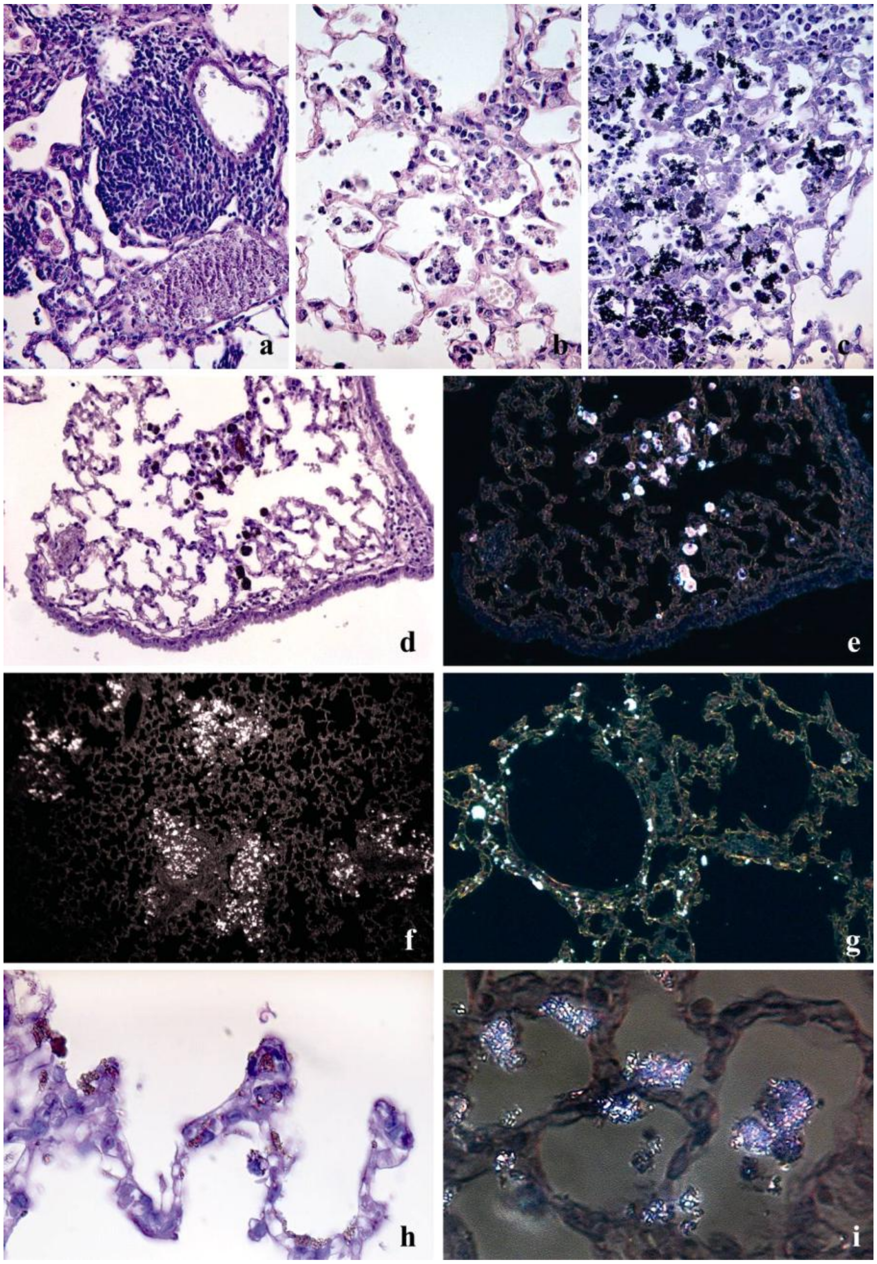

**FIGURE 3.** Representative histological sections of lungs from mice that had received a single i.t. instillation of quartz (SRM1878a or DQ12) or an instillation of a species of $TiO_2$. The animals showed different inflammatory changes as well as deposition of particles. (a) Three months postinstillation, subchronic infiltration of inflammatory cells close to two vascular structures in a DQ12-treated mouse. (b) Focal acute inflammation in the alveoli 24 h after i.t. instillation of SRM1878a. (c) Plenty of macrophages in the alveoli containing black particles in a mouse treated 24 h previously with $TiO_2$MR. (d and e) Three months post-treatment of a mouse with $TiO_2$NAM; the section was photographed using bright- and dark-field microscopy, respectively. The macrophages are easily identified. (f) A somewhat uneven distribution of micro-size rutile particles 24 h postinstillation, demonstrated at low magnification and dark-field microscopy. (g) Particles of $TiO_2$NAM 24 h postinstillation were mainly in the interstitial tissue and not in macrophages. (h) $TiO_2$NAM 24 h postinstillation are accumulated in macrophages, but are also seen directly on the surface of the alveoli. (i) Larger magnification of the same exposure as in (h) using polarization microscopy. Macrophages containing particles are seen in the lumen of the alveoli. The resolution is superior compared to the dark-field micrographs. Staining hematoxylin/PAS, magnifications: a, b, and c: ×150; d and e: ×60; f: ×25; g: ×250; h: ×480; and i: ×600.

**TABLE 2**
**Summary of Histological Findings in the Lungs after a Single Intratracheal Instillation in Mice, Studied after 24 h and 3 Months**

| Particles | 24 h | | | 3 months | | |
|---|---|---|---|---|---|---|
| | 5 µg | 50 µg | 500 µg | 5 µg | 50 µg | 500 µg |
| 1878a | 0 | 0 | x | 0 | 0 | xx |
| DQ12 | 0 | 0 | x | 0 | 0 | x |
| $TIO_2MR$ | xx | xx | xxxxx | 0 | 0 | x |
| $TIO_2NR$ | x | xxx | xxxx | 0 | x | xxx |
| $TIO_2NA$ | 0 | x | xxx | 0 | 0 | x |
| $TIO_2NAM$ | x | xxx | xxxx | x | xx | xxx |
| **Inflammation** | **24 h** | | | **3 months** | | |
| | **5 µg** | **50 µg** | **500 µg** | **5 µg** | **50 µg** | **500 µg** |
| 1878a | 0 | 0 | x | 0 | 0 | xx |
| DQ12 | 0 | 0 | x | 0 | 0 | x |
| $TIO_2MR$ | 0 | x | xxx | 0 | 0 | 0 |
| $TIO_2NR$ | 0 | 0 | x | 0 | 0 | x |
| $TIO_2NA$ | 0 | 0 | x | 0 | 0 | 0 |
| $TIO_2NAM$ | 0 | 0 | 0 | 0 | 0 | 0 |

The level of visible particles and inflammation in the lungs were scored with 0–5 Xs.

### Quartz – Inflammatory Cells in BALF

For both types of quartz, a dose-dependent increase was seen for neutrophils in BALF in the acute phase (Fig. 4a) that was similar for both types of quartz. After 3 months, the level of neutrophils was reduced, although still significantly and dose dependently increased.

In the acute phase, macrophages in BALF showed a minor, and in one case, a statistically significant, decrease. However, these changes were not dose dependent (Fig. 4b) and may be a spurious finding. However, because we also found a single significant decrease in the acute phase in our previous study[14], the results may be real. The macrophage level is a result of removal and infiltration, and minor changes may be complex to interpret. Nevertheless, it stands out that no major change in macrophage levels occurs in the acute phase. After 3 months, a dose-dependent increase of macrophages was observed only for DQ12. No significant effect on lymphocytes, eosinophils, or epithelial cells was seen (data not shown).

### Quartz – Biochemical Markers in BALF

In the acute phase, instillation of both types of quartz resulted in a significant increase of IL-6 at the highest dose (Fig. 5). The levels were close to the control levels 3 months after instillation.

Total protein in BALF showed a dose-dependent increase after exposure to both types of quartz, being highest for DQ12 (Fig. 6). Three months after instillation, levels were similar to the control levels. The levels of TNF-α were above the LOD in only six out of 384 samples and no dose-dependent effect was apparent (data not shown).

a)

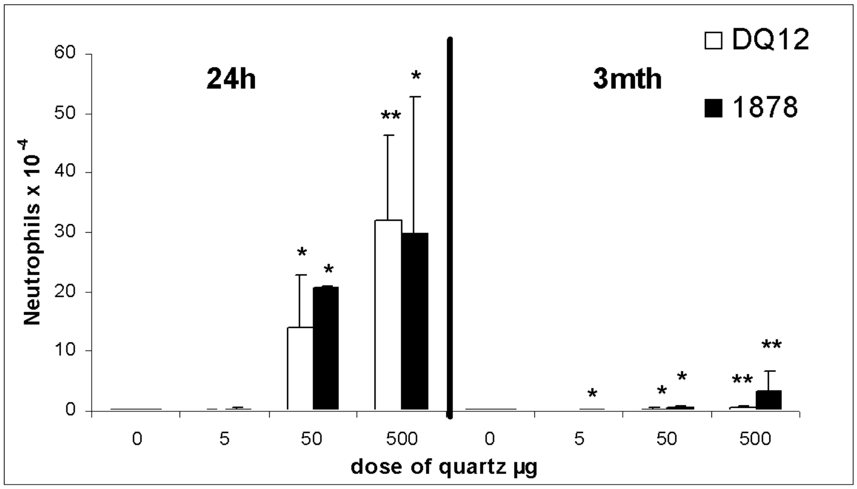

b)

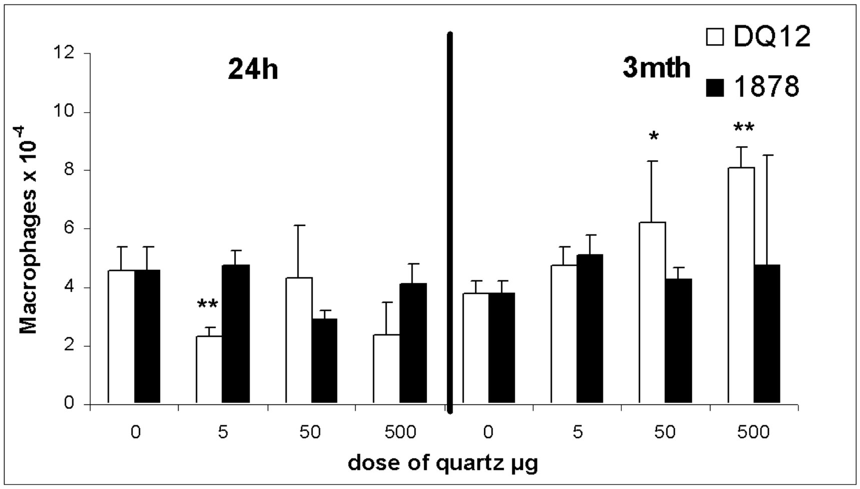

**FIGURE 4.** Quartz - inflammatory cells in BALF. BALF was collected 24 h or 3 months after a single i.t. instillation of DQ12 (white) or SRM1878a (black) in BALB/c mice. The (a) neutrophil and (b) macrophage content in BALF. Number of cells is shown as the median and 75th percentile. For explanation of statistical test results (* and **), see Statistics. Each group contained eight mice.

### Quartz – Histology

One day after instillation of quartz, a low number of particles appeared from light microscopy (Table 2) that seemed not to decrease over the succeeding 3-month period.

A weak lung inflammation was observed for both types of quartz, both in the acute and the subchronic phase.

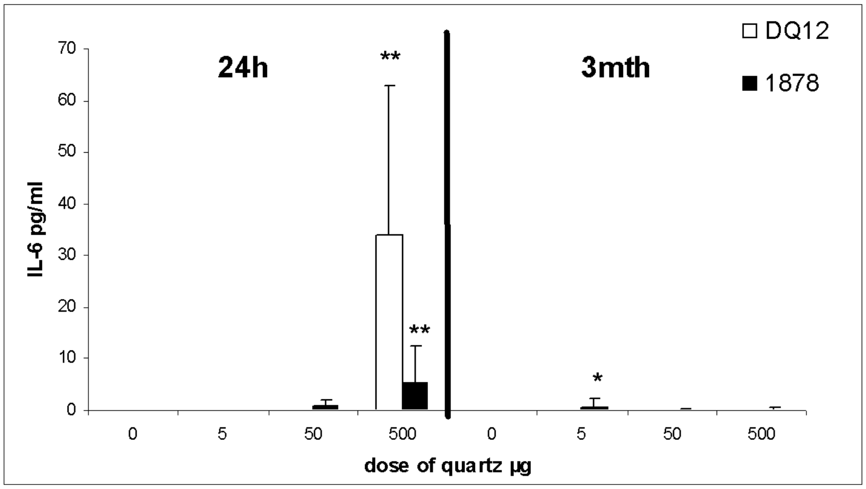

**FIGURE 5.** Quartz - IL-6 in BALF. The levels of IL-6 in BALF are shown as the median and 75th percentile for DQ12 (white) and SRM1878a (black). BALF was collected 24 h or 3 months postinstillation in BALB/c mice. For explanation of statistical test results (* and **), see Statistics. Each group contained eight mice.

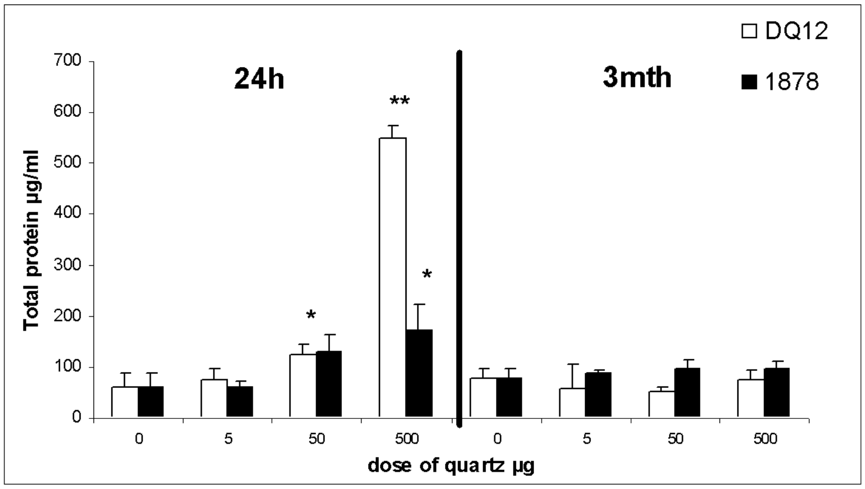

**FIGURE 6.** Quartz - total protein in BALF. The levels of total protein in BALF are shown as the median and 75th percentile for DQ12 .(white) and SRM1878a (black). BALF was collected 24 h or 3 months postinstillation in BALB/c mice. For explanation of statistical test results (* and **), see Statistics. Each group contained eight mice.

### $TiO_2$ – Inflammatory Cells in BALF

For the four types of $TiO_2$ particles, a conspicuous influx of neutrophils was observed in the acute phase (Fig. 7a) that was maximal at the highest dose where all fluxes were significantly increased compared with controls. The highest levels were reached after exposure to the rutile $TiO_2$ and independent of the particle size. After 3 months, the levels were still significantly different from the controls, but close to these levels.

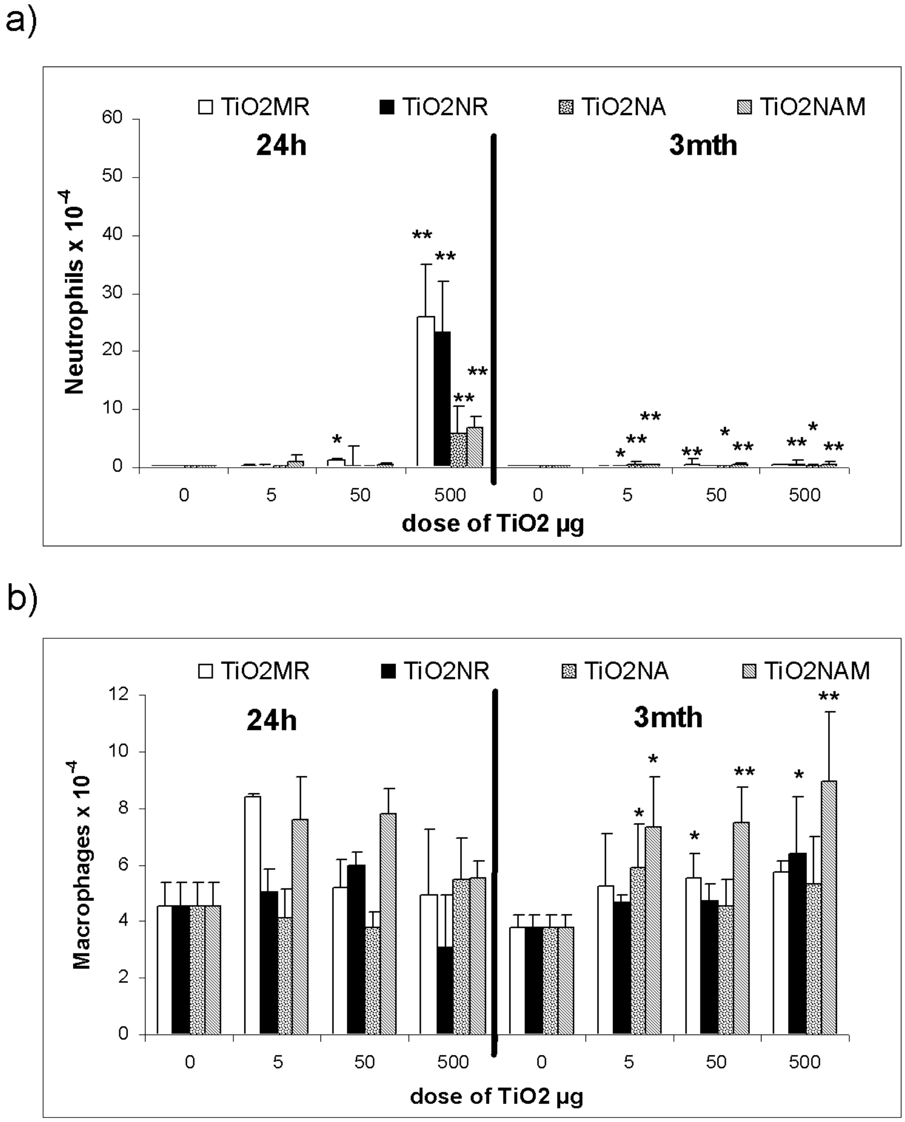

**FIGURE 7**. $TiO_2$ - inflammatory cells in BALF. Collection of BALF was performed 24 h or 3 months after a single i.t. instillation of $TiO_2$ for micro-size rutile (MR - white), nano-size rutile (NR - black), nano-size anatase (NA - dotted), and nano-size amorphous (NAM - dashed) in BALB/c mice. (a) Neutrophils; (b) macrophages in BALF. Number of cells is shown as the median and 75$^{th}$ percentile. For explanation of statistical test results (* and **), see Statistics. Each group contained eight mice.

For macrophages, no dose-dependent effect was seen in the acute phase (Fig. 7b). After 3 months, the amorphous $TiO_2$ significantly induced elevated macrophage levels at all doses. Nano-size rutile $TiO_2$ induced a significant increase in macrophages at the highest dose level, suggesting a dose-dependent effect. No dose-dependent effect was seen for the other particles. No effect was seen on lymphocytes, eosinophils, or epithelial cells (data not shown).

### $TiO_2$ – Biochemical Markers in BALF

Except for the anatase particles, IL-6 showed a transient increase in the acute phase (Fig. 8), although only significant for the nano-size rutile $TiO_2$. After 3 months, the levels were close to control levels and no dose-dependent effect was apparent. In the acute phase, the level of total protein in BALF was increased at the highest dose of all particles except the anatase $TiO_2$. The highest level was observed in the group exposed to amorphous $TiO_2$ (Fig. 9). After 3 months, no dose-dependent increase was observed in any of the groups. None of the exposures increased TNF-α, neither in the acute nor in the subchronic phase (data not shown).

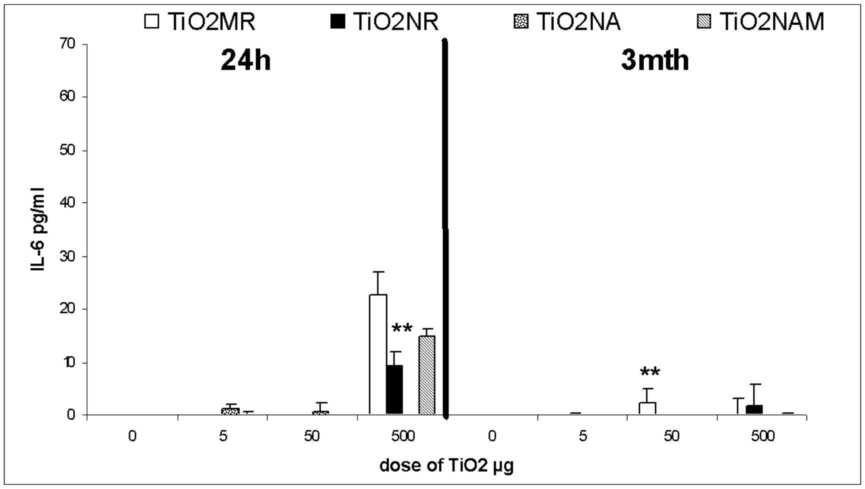

**FIGURE 8.** $TiO_2$ - IL-6 in BALF. The levels of IL-6 in BALF are shown as the median and 75$^{th}$ percentile for micro-size rutile (MR - white), nano-size rutile (NR - black), nano-size anatase (NA - dotted), and nano-size amorphous (NAM - dashed). BALF was collected 24 h or 3 months postinstillation in BALB/c mice. For explanation of statistical test results (**), see Statistics. Each group contained eight mice.

### $TiO_2$ – Histology

One day after instillation, all types of $TiO_2$ showed a dose-dependent deposition in the lungs by dark-field and polarization microscopy (Table 2). The identified depositions were roughly similar at similar dose levels, and independent of particle sizes. After 3 months, the visible lung burden of micro-size rutile and nano-size anatase $TiO_2$ had decreased substantially, whereas particles were still visible in high numbers for the nano-size rutile (data not shown) and the nano-size amorphous $TiO_2$ (Fig. 3d and e). The most pronounced inflammatory changes were seen 1 day after instillation of micro-size rutile $TiO_2$ (Fig. 3c), which correlates with the high visible lung burden of particles. After 3 months, the inflammation and the visible particles had disappeared. Nano-size rutile, anatase, and amorphous $TiO_2$ showed very little histological inflammation, although the visible lung burden was high in the acute phase (e.g., Fig. 3g–i). In the subchronic phase, a relatively high lung burden was apparent for nano-size rutile (data not shown) and amorphous $TiO_2$ (Fig. 3d,e), but only the nano-size rutile–exposed group showed a slight lung inflammation.

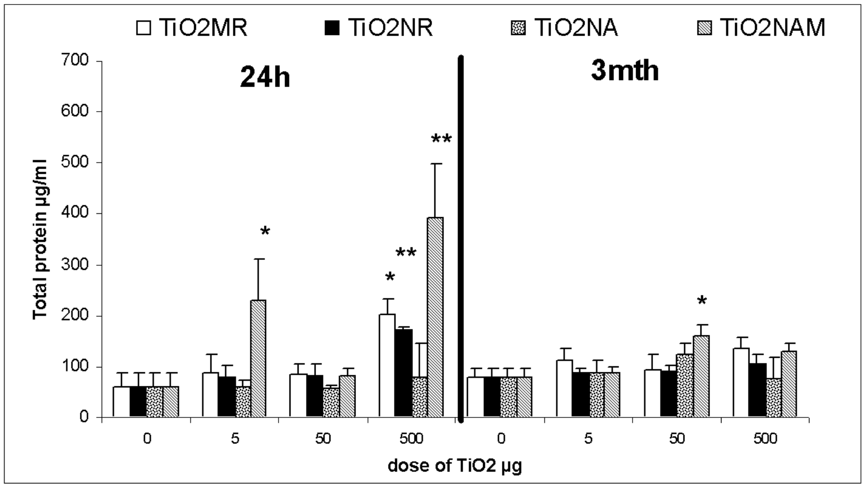

**FIGURE 9.** $TiO_2$ - total protein in BALF. The levels of total protein in BALF are shown as the median and 75th percentile for micro-size rutile (MR - white), nano-size rutile (NR - black), nano-size anatase (NA - dotted), and nano-size amorphous (NAM - dashed). BALF was collected 24 h or 3 months postinstillation in BALB/c mice. For explanation of statistical test results (* and **), see Statistics. Each group contained eight mice.

### Exposure Metrics and Potency of Acute Neutrophil Inflammation

To assess the potential relevance of surface area–driven toxicity, the inflammatory responses were plotted against the instilled surface area of particles per mouse. In this study, the two micro-size quartz and the micro-size rutile particles had the highest potency to cause influx of neutrophils (Fig. 10a) and elevating levels of total protein in BALF in the acute phase (data not shown). An intermediate effect was apparent for nano-size rutile, whereas the effects were lowest for nano-size anatase and amorphous $TiO_2$. Taking the high degree of agglomeration into account, a comparison was also made with a theoretical instilled agglomerate surface area as estimated from the hydrodynamic diameters ($d_H$) obtained by DLS analysis. Again the two types of quartz had the highest potency, showing influx of neutrophils both at the middle and the high dose. A high influx of neutrophils occurred only at the highest dose of nano- and micro-size rutile $TiO_2$, indicating a lower potency for these particles. As the neutrophil influx was low at the highest dose of nano-size anatase and amorphous $TiO_2$, these particles had the lowest potency (Fig. 10b). The same trends were seen for the mass metric as from the surface area estimated from the $d_H$ (Fig. 10c). Irrespective of the metrics, the highest potency was observed for quartz, an intermediate potency for rutile particles irrespective of the particle sizes, and the lowest potency was always observed for nano-size anatase and amorphous $TiO_2$.

### DISCUSSION

The purpose of this study was to compare the dose-response relations of different µm- and nano-size quartz and $TiO_2$ particles in regard to their inflammatory effect in mice. Three doses of six different particles were instilled into the lungs of mice, and inflammation was assessed both 24 h and 3 months postexposure. We used the two types of reference quartz to compare particle size effects of $TiO_2$ as well as the potential different effects of different $TiO_2$ polymorphs.

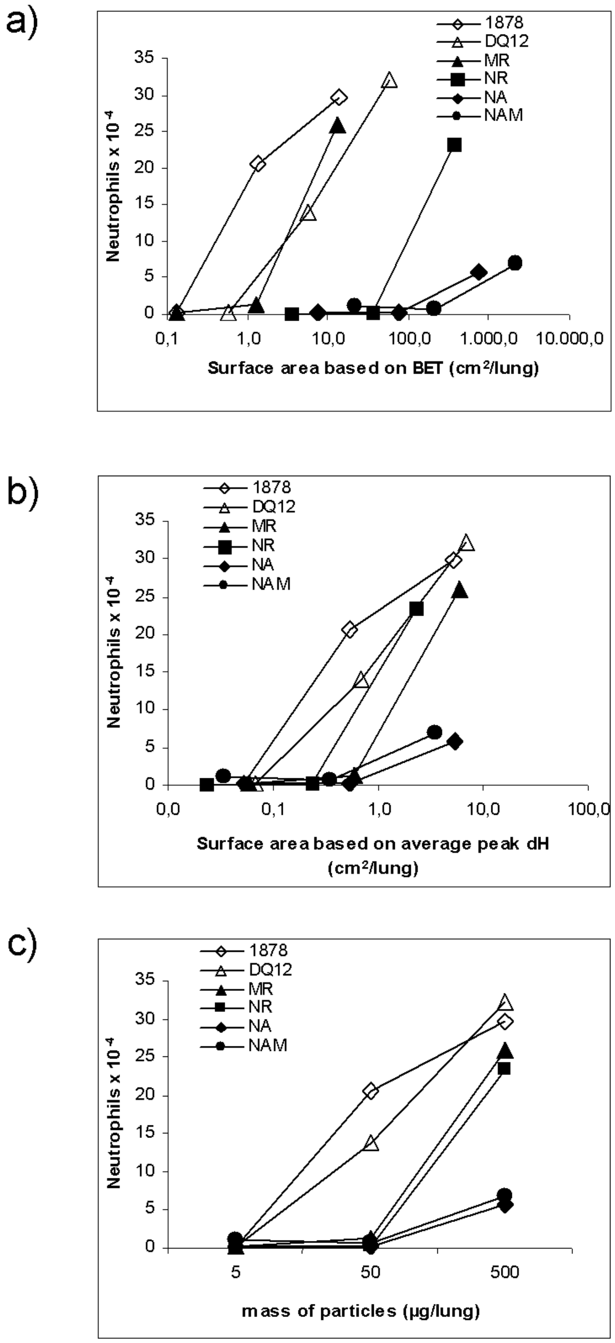

**FIGURE 10.** Surface area and mass vs. acute neutrophil inflammation. The surface area and the mass instilled for each type of particle were plotted against neutrophil counts 24 h postexposure. (a) The particle surface areas instilled were calculated from the BET size and (b) from the hydrodynamic diameter (dH). (c) The mass of instilled particles was also used as metrics.

### Quartz – Comparison of SRM1878a and DQ12

To our knowledge, this is the first study comparing the inflammatory effect of SRM1878a and DQ12. Although both types of quartz are often used as benchmark materials, it has been difficult to compare results across studies due to lack of a scaling factor, accounting for differences in toxicities of the different compounds. Also, data from low-dose exposures are scarce. The acute inflammatory profiles were roughly similar for the two types of instilled quartz, irrespective of the use of the mass or the surface area metrics. Both types showed a slight neutrophil inflammation in the subchronic phase. However, in relation to the subchronic effect, DQ12 seems to be more potent than the SRM1878a on the macrophage response. In part, this could be a consequence of the seemingly smaller particle size of the DQ12 quartz as seen from both BET, DLS, and TEM (Table 1).

### $TiO_2$ – Effect of Particle Size

The acute and subchronic effects of instilled micro- and nano-size rutile $TiO_2$ showed similar effects on the neutrophil and the macrophage response, and on IL-6 and total protein in BALF. Micro-size rutile $TiO_2$ showed a more pronounced acute lung inflammation by histology, but no inflammation was observed after 3 months, where a slight inflammation was still observed for the nano-size rutile $TiO_2$. The inflammation paralleled the visible lung burden of particles. Overall, the two sizes of rutile $TiO_2$ had roughly similar inflammatory effects. The similar potency of the rutile $TiO_2$ could indicate that the agglomeration size is important for the effect, which could explain the contradicting effects found in other studies, showing higher effect for larger particles[35,49,50] or higher effect of nano-size particles[40,42,43,51,52]. In our study, the DLS data indicate that the two sizes of rutile $TiO_2$ particles in solution had approximately the same agglomerate size; why the biological defense system may be able to identify and handle the materials similarly with the same exerted biological effect.

Surface area has been shown to be a predictor for the inflammatory effects of low soluble and low toxic particles[40,42,43,51], but other factors may have an equal or greater importance. Factors suggested to influence biological effects include the agglomeration state, the vehicle, and the exposure route used, as well as surface coatings[13,23,24,41,49,53,54,55]. A difference in agglomerate size in vehicles may affect the deposition in the lungs as well as the clearance efficiency, and thus determine whether or not the particles reach the alveolar region[40,49,56]. The powder surface area was measured with BET nitrogen adsorption and the hydrodynamic agglomerate surface area was estimated from DLS analysis. Overall, plotting against the two types of measures showed a similar ranking of inflammatory potencies, i.e., quartz $\geq$ rutile $TiO_2$ > anatase and amorphous $TiO_2$. Despite this, the dose-response curves were grouped closer when using the hydrodynamic agglomerate surface area than BET surface area or mass; material-specific effects still appeared to dominate the biological response within this set of test materials.

One reason for the different potencies from different studies could be that the degree of agglomeration of particles is difficult to evaluate. Often, the mass or the surface area metric are compared in evaluation of inflammatory effects, where the surface area is measured by BET analysis on the dry particles. When suspending particles in vehicles, agglomeration may result in an assembly of particles where the biologically effective surface area is lower than the BET surface area. In fact, similar agglomeration size, especially when the size is within the range enabling efficient phagocytosis and deagglomeration does not occur in the lung, may cause a similar clearance. Thus, a biological system may interact with agglomerates with physical properties different from these of their single particles. Therefore, it might also be highly relevant to characterize the particles in the specific vehicle in order to gain insight into the degree of agglomeration that is actually encountered by the airways, and to investigate further whether deagglomeration takes place in the airways.

### $TiO_2$ – Effect of Polymorphism

The polymorphism of a compound has been shown to be important for its biological effect. For example, Sayes et al. found for nano-size $TiO_2$ in an *in vitro* study that anatase was two times more toxic than rutile $TiO_2$[35], which is in contrast to our results; the rutile $TiO_2$ used in their study was reported to be highly aggregated. In another study by Lu et al., investigating the lung influx of neutrophils in rats, the result was similar to ours, where the influx of neutrophils was most prominent for rutile $TiO_2$ and less prominent for anatase[57]. In our study, the rutile form of $TiO_2$ was the most inflammogenic of the $TiO_2$ particles based on the acute influx of neutrophils and histology (Fig. 7a and Table 2), whereas the amorphous $TiO_2$ was the most potent particle to induce increase in the level of total protein in BALF (Fig. 9) and subchronic accumulation of macrophages in BALF (Fig. 7b). From electron microscopy, nano-size rutile and amorphous $TiO_2$ both appeared as being instilled in more open or loosely agglomerated particles than anatase (Fig. 2). This morphological difference may be of importance and play a role in the potential deagglomeration in the airways. Unfortunately, further analysis was not possible due to lack of material.

In addition to the differences in polymorphism, surface properties also played a role in the biological effects[13,51,55,58], which can be one of the reasons for the somewhat contradictory findings in different studies. The surface properties and physical state of the particles may vary depending on manufacturing processes. In this case, only the micro-size rutile was produced by flame synthesis. The other titanium materials were produced by wet-chemical synthesis and subsequent calcination. The effect of such differences should be further investigated.

For the nano-size particles in this study, little visible anatase $TiO_2$ was apparent in the lungs after 3 months, whereas plenty of visible rutile and amorphous $TiO_2$ was observed (Table 2 and Fig. 3e), and inflammation was observed by increased neutrophil and macrophage counts in BALF (Fig. 7). Histology suggested that amorphous $TiO_2$ was not inflammogenic, neither in the acute nor in the subchronic phase, whereas the rutile micro-size particles were highly inflammogenic in the acute phase (Fig. 3c) with plenty of visible particles. After 3 months, no indications of inflammation were observed for the micro-size rutile $TiO_2$ and no visible particles were apparent. The histological findings in regard to the presence of particles should be interpreted as qualitative or indicative, as the scoring of particles in the lungs was performed after the lungs were flushed for collection of the BALF, but comparisons should be valid.

### Quartz vs. $TiO_2$

The inflammatory potency of quartz was significantly higher than that of $TiO_2$ based on the influx of neutrophils. Exposure to 50 μg of either type of quartz resulted in a high influx of neutrophils (Fig. 4a), where a dose of 500 μg was needed for the $TiO_2$ particles (except micro-size rutile) to induce an influx (Fig. 7a). This is consistent with other studies using quartz as a positive control for investigation of inflammatory effects of different types of $TiO_2$[13,50,59,60,61].

### Comparison of Metrics

A major discussion in the nanotoxicology field is what dose metrics should be used when interpreting data. Thus, we have presented our data using both particle mass as well as the surface area as metrics. The surface area metrics were obtained from either the “dry” particles (based on BET data) or from the “wet” particles where the surface area has been calculated from the DLS data. The BET surface may be used as a metric for comparison of inflammatory effects. The BET surface area is obtained from gas adsorption onto dry particles, allowing determination of the entire surface area of particles. Using this metrics, it is interesting that the micro-size particles of both types of quartz and the micro-size rutile $TiO_2$ induce comparable acute neutrophilic influx at the highest dose (Fig. 10a), suggesting an approximately similar potency per surface area if it is assumed that the BET surface is an appropriate estimate of the biologically relevant surface. However, if the biologically accessible surface area is less for the nano-size materials due to agglomeration, the biologically relevant surface is overestimated and thus the potency will be underestimated for these materials.

When calculating the surface area from the $d_H$ obtained from the DLS data, the results indicate that the inflammatory effect of the two types of quartz and the two types of rutile $TiO_2$ induced a comparable acute neutrophil inflammation at the highest dose, whereas the effect of the anatase and amorphous $TiO_2$ was significantly lower (Fig. 10b). If the biologically accessible surface area of the nanoparticles from DLS is underestimated, the potency of the particles will be overestimated. However, as potencies based both on the area from the BET and the DLS analyses show that nano-size anatase and amorphous $TiO_2$ have lower potencies than the quartz and the rutile compounds, this can be considered a definite conclusion from this study. The mass metrics rank the potencies of the compounds equal to the two surface metrics and thus provide a relatively robust estimate in the present case.

A low level of inflammation in the tissue was still seen 3 months after instillation of both types of quartz and for the nano-size rutile $TiO_2$ (Table 2). This was consistent with the unaltered content of particles in the lungs. For the amorphous $TiO_2$, visible particles were prominent both in the acute phase and after 3 months, but inflammation in the tissue was very low, indicating a low inflammogenic effect, although macrophage response was still apparent after 3 months. Overall, the inflammogenic potential depends on the studied end point and the type of particle surface.

### CONCLUSIONS

The two types of quartz had roughly similar inflammatory effects, except for the subchronic macrophage response, indicating a possible chronic inflammation that may be more prominent with DQ12. This suggests that potencies of particles obtained with SRM1878a and DQ12 as benchmark compounds are roughly independent of the used reference compound, except for the subchronic macrophage response.

No significant differences were found between the two sizes of rutile $TiO_2$, but clear differences were seen between the different forms of nano-size $TiO_2$, with rutile as the most inflammogenic type of $TiO_2$ and amorphous as the most potent in relation to tissue damage based on the level of total protein in BALF.

Our characterization data suggest that emphasis should be put into characterizing the particles in the vehicle used for the exposure as well as a better understanding of particle reactivity. The level of agglomeration of particles may have a significant impact on the toxicity, as the physical properties of the agglomerates may be of importance for the deposition patterns, for their interaction with the target area and efficiency of the biological defense mechanisms enabling phagocytosis, and preventing endocytosis and general particle translocation. In our study, the use of surface area calculated from the hydrodynamic diameter and the mass metric showed approximately similar toxicological profiles of the inflammatory effects of the tested compounds.

### ACKNOWLEDGMENTS

This work was supported through the NANOKEM project funded by the National Working Environment Fund. All the nano-size $TiO_2$ particles were a kind gift from Professor A. Navrotsky (University of California at Davis, California, U.S.A.) and were synthesized and analyzed by Ranade et al.[44] for their water and carbon content, their specific surface area, powder X-ray crystallography and crystallite size by Rietveld analysis, and calorimetry. Heidi Maria Paulsen and Lise Strange are thanked for the histological preparation.

## References

1. Donaldson, K. and Borm, P.J. (1998) The quartz hazard: a variable entity. *Ann. Occup. Hyg.* **42,** 287–294.
2. Merget, R., Bauer, T., Kupper, H.U., Philippou, S., Bauer, H.D., Breitstadt, R., and Bruening, T. (2002) Health hazards due to the inhalation of amorphous silica. *Arch. Toxicol.* **75,** 625–634.
3. Parr, D.G., White, A.J., Bayley, D.L., Guest, P.J., and Stockley, R.A. (2006) Inflammation in sputum relates to progression of disease in subjects with COPD: a prospective descriptive study. *Respir. Res.* **7,** 136.
4. Attfield, M.D. (1997) IARC Library Cataloguing in Publication Data. pp. 1-521.
5. Alessandrini, F., Schulz, H., Takenaka, S., Lentner, B., Karg, E., Behrendt, H., and Jakob, T. (2006) Effects of ultrafine carbon particle inhalation on allergic inflammation of the lung. *J. Allergy Clin. Immunol.* **117,** 824–830.
6. Larsen, S.T., Roursgaard, M., Jensen, K.A., and Nielsen, G.D. (2010) Nano titanium dioxide particles promote allergic sensitization and lung inflammation in mice. *Basic Clin. Pharmacol. Toxicol.* **106,** 114–117.
7. Nygaard, U.C., Hansen, J.S., Samuelsen, M., Alberg, T., Marioara, C.D., and Lovik, M. (2009) Single-walled and multi-walled carbon nanotubes promote allergic immune responses in mice. *Toxicol. Sci.* **109,** 113–123.
8. Donaldson, K., Stone, V., Gilmour, P.S., Brown, D.M., and MacNee, W. (2000) Ultrafine particles: mechanisms of lung injury. *Philos. Trans. R. Soc. Lond. Ser. A* **358,** 2741–2748.
9. Borm, P.J., Robbins, D., Haubold, S., Kuhlbusch, T., Fissan, H., Donaldson, K., Schins, R., Stone, V., Kreyling, W., Lademann, J., et al. (2006) The potential risks of nanomaterials: a review carried out for ECETOC. *Part. Fibre Toxicol.* **3,** 11.
10. Renwick, L.C., Brown, D., Clouter, A., and Donaldson, K. (2004) Increased inflammation and altered macrophage chemotactic responses caused by two ultrafine particle types. *Occup. Environ. Med.* **61,** 442–447.
11. Kajiwara, T., Ogami, A., Yamato, H., Oyabu, T., Morimoto, Y., and Tanaka, I. (2007) Effect of particle size of intratracheally instilled crystalline silica on pulmonary inflammation. *J. Occup. Health* **49,** 88–94.
12. Oberdorster, G. (2001) Pulmonary effects of inhaled ultrafine particles. *Int. Arch. Occup. Environ. Health* **74,** 1–8.
13. Warheit, D.B., Webb, T.R., Reed, K.L., Frerichs, S., and Sayes, C.M. (2007) Pulmonary toxicity study in rats with three forms of ultrafine-TiO2 particles: differential responses related to surface properties. *Toxicology* **230,** 90–104.
14. Roursgaard, M., Poulsen, S.S., Poulsen, L.K., Hammer, M., Jensen, K.A., Utsunomiya, S., Ewing, R.C., Balic-Zunic, T., Nielsen, G.D., and Larsen, S.T. (2010) Time-response relationship of nano and micro particle induced lung inflammation. Quartz as reference compound. *Hum. Exp. Toxicol.* **29,** 915–933.
15. Samuelsen, M., Nygaard, U.C., and Lovik, M. (2009) Particle size determines activation of the innate immune system in the lung. *Scand. J. Immunol.* **69,** 421–428.
16. Oberdorster, G., Maynard, A., Donaldson, K., Castranova, V., Fitzpatrick, J., Ausman, K., Carter, J., Karn, B., Kreyling, W., Lai, D., et al. (2005) Principles for characterizing the potential human health effects from exposure to nanomaterials: elements of a screening strategy. *Part. Fibre Toxicol.* **2,** 8.
17. Warheit, D.B., Borm, P.J., Hennes, C., and Lademann, J. (2007) Testing strategies to establish the safety of nanomaterials: conclusions of an ECETOC workshop. *Inhal. Toxicol.* **19,** 631–643.
18. Ma-Hock, L., Burkhardt, S., Strauss, V., Gamer, A.O., Wiench, K., van Ravenzwaay, B., and Landsiedel, R. (2009) Development of a short-term inhalation test in the rat using nano-titanium dioxide as a model substance. *Inhal. Toxicol.* **21,** 102–118.
19. Sayes, C.M., Reed, K.L., and Warheit, D.B. (2007) Assessing toxicity of fine and nanoparticles: comparing in vitro measurements to in vivo pulmonary toxicity profiles. *Toxicol. Sci.* **97,** 163–180.
20. Maynard, A.D., Aitken, R.J., Butz, T., Colvin, V., Donaldson, K., Oberdorster, G., Philbert, M.A., Ryan, J., Seaton, A., Stone, V., et al. (2006) Safe handling of nanotechnology. *Nature* **444,** 267–269.
21. Seagrave, J., McDonald, J.D., and Mauderly, J.L. (2005) In vitro versus in vivo exposure to combustion emissions. *Exp. Toxicol. Pathol.* **57(Suppl 1),** 233–238.
22. Jacobsen, N.R., Moller, P., Jensen, K.A., Vogel, U., Ladefoged, O., Loft, S., and Wallin, H. (2009) Lung inflammation and genotoxicity following pulmonary exposure to nanoparticles in ApoE-/- mice. *Part. Fibre Toxicol.* **6,** 2.
23. Osier, M., Baggs, R.B., and Oberdorster, G. (1997) Intratracheal instillation versus intratracheal inhalation: influence of cytokines on inflammatory response. *Environ. Health Perspect.* **105(Suppl 5)**, 1265–1271.
24. Osier, M. and Oberdorster, G. (1997) Intratracheal inhalation vs intratracheal instillation: differences in particle effects. *Fundam. Appl. Toxicol.* **40,** 220–227.
25. Warheit, D.B., Brock, W.J., Lee, K.P., Webb, T.R., and Reed, K.L. (2005) Comparative pulmonary toxicity inhalation and instillation studies with different TiO2 particle formulations: impact of surface treatments on particle toxicity. *Toxicol. Sci.* **88,** 514–524.
26. Henderson, R.F., Driscoll, K.E., Harkema, J.R., Lindenschmidt, R.C., Chang, I.Y., Maples, K.R., and Barr, E.B. (1995) A comparison of the inflammatory response of the lung to inhaled versus instilled particles in F344 rats. *Fundam. Appl. Toxicol.* **24,** 183–197.
27. Pritchard, J.N., Holmes, A., Evans, J.C., Evans, N., Evans, R.J., and Morgan, A. (1985) The distribution of dust in the rat lung following administration by inhalation and by single intratracheal instillation. *Environ. Res.* **36,** 268–297.

28. Albrecht, C., Schins, R.P., Hohr, D., Becker, A., Shi, T., Knaapen, A.M., and Borm, P.J. (2004) Inflammatory time course after quartz instillation: role of tumor necrosis factor-alpha and particle surface. *Am. J. Respir. Cell Mol. Biol.* **31,** 292–301.
29. Warheit, D.B., Webb, T.R., Colvin, V.L., Reed, K.L., and Sayes, C.M. (2007) Pulmonary bioassay studies with nanoscale and fine-quartz particles in rats: toxicity is not dependent upon particle size but on surface characteristics. *Toxicol. Sci.* **95,** 270–280.
30. Duffin, R., Gilmour, P.S., Schins, R.P., Clouter, A., Guy, K., Brown, D.M., MacNee, W., Borm, P.J., Donaldson, K., and Stone, V. (2001) Aluminium lactate treatment of DQ12 quartz inhibits its ability to cause inflammation, chemokine expression, and nuclear factor-kappaB activation. *Toxicol. Appl. Pharmacol.* **176,** 10–17.
31. Lam, C.W., James, J.T., McCluskey, R., and Hunter, R.L. (2004) Pulmonary toxicity of single-wall carbon nanotubes in mice 7 and 90 days after intratracheal instillation. *Toxicol. Sci.* **77,** 126–134.
32. Arts, J.H., Muijser, H., Duistermaat, E., Junker, K., and Kuper, C.F. (2007) Five-day inhalation toxicity study of three types of synthetic amorphous silicas in Wistar rats and post-exposure evaluations for up to 3 months. *Food Chem. Toxicol.* **45,** 1856–1867.
33. van Ravenzwaay, B., Landsiedel, R., Fabian, E., Burkhardt, S., Strauss, V., and Ma-Hock, L. (2009) Comparing fate and effects of three particles of different surface properties: nano-TiO(2), pigmentary TiO(2) and quartz. *Toxicol. Lett.* **186,** 152–159.
34. Borm, P.J. and Kreyling, W. (2004) Toxicological hazards of inhaled nanoparticles--potential implications for drug delivery. *J. Nanosci. Nanotechnol.* **4,** 521–531.
35. Sayes, C.M., Wahi, R., Kurian, P.A., Liu, Y., West, J.L., Ausman, K.D., Warheit, D.B., and Colvin, V.L. (2006) Correlating nanoscale titania structure with toxicity: a cytotoxicity and inflammatory response study with human dermal fibroblasts and human lung epithelial cells. *Toxicol. Sci.* **92,** 174–185.
36. Bermudez, E., Mangum, J.B., Wong, B.A., Asgharian, B., Hext, P.M., Warheit, D.B., and Everitt, J.I. (2004) Pulmonary responses of mice, rats, and hamsters to subchronic inhalation of ultrafine titanium dioxide particles. *Toxicol. Sci.* **77,** 347–357.
37. Morrow, P.E. (1988) Possible mechanisms to explain dust overloading of the lungs. *Fundam. Appl. Toxicol.* **10,** 369–384.
38. Monteiller, C., Tran, L., MacNee, W., Faux, S., Jones, A., Miller, B., and Donaldson, K. (2007) The pro-inflammatory effects of low-toxicity low-solubility particles, nanoparticles and fine particles, on epithelial cells in vitro: the role of surface area. *Occup. Environ. Med.* **64,** 609–615.
39. Donaldson, K., Brown, D., Clouter, A., Duffin, R., MacNee, W., Renwick, L., Tran, L., and Stone, V. (2002) The pulmonary toxicology of ultrafine particles. *J. Aerosol Med.* **15,** 213–220.
40. Ferin, J., Oberdorster, G., and Penney, D.P. (1992) Pulmonary retention of ultrafine and fine particles in rats. *Am. J. Respir. Cell Mol. Biol.* **6,** 535–542.
41. Oberdorster, G. (1996) Significance of particle parameters in the evaluation of exposure-dose-response relationships of inhaled particles. *Inhal. Toxicol.* **8(Suppl),** 73–89.
42. Oberdorster, G., Ferin, J., and Lehnert, B.E. (1994) Correlation between particle size, in vivo particle persistence, and lung injury. *Environ. Health Perspect.* 1**02(Suppl 5),** 173–179.
43. Tran, C.L., Buchanan, D., Cullen, R.T., Searl, A., Jones, A.D., and Donaldson, K. (2000) Inhalation of poorly soluble particles. II. Influence of particle surface area on inflammation and clearance. *Inhal. Toxicol.* **12,** 1113–1126.
44. Ranade, M.R., Navrotsky, A., Zhang, H.Z., Banfield, J.F., Elder, S.H., Zaban, A., Borse, P.H., Kulkarni, S.K., Doran, G.S., and Whitfield, H.J. (2002) Energetics of nanocrystalline TiO2. *Proc. Natl. Acad. Sci. U. S. A.* **99(Suppl 2),** 6476–6481.
45. Mukhopadhyay, S., Hoidal, J.R., and Mukherjee, T.K. (2006) Role of TNFalpha in pulmonary pathophysiology. *Respir. Res.* **7,** 125.
46. Orihara, K. and Matsuda, A. (2008) Pathophysiological roles of microvascular alterations in pulmonary inflammatory diseases: possible implications of tumor necrosis factor-alpha and CXC chemokines. *Int. J. Chron. Obstruct. Pulmon. Dis.* **3,** 619–627.
47. Larsen, S.T., Hansen, J.S., Hammer, M., Alarie, Y., and Nielsen, G.D. (2004) Effects of mono-2-ethylhexyl phthalate on the respiratory tract in BALB/c mice. *Hum. Exp. Toxicol.* **23,** 537–545.
48. Crawford, J.R. and Garthwaite, P.H. (2009) Percentiles please: the case for expressing neuropsychological test scores and accompanying confidence limits as percentile ranks. *Clin. Neuropsychol.* **23,** 193–204.
49. Grassian, V.H., Adamcakova-Dodd, A., Pettibone, J.M., O'Shaughnessy, P.T., and Thorne, P.S. (2007) Inflammatory response of mice to manufactured titanium dioxide nanoparticles: comparison of size effects through different exposure routes. *Nanotoxicology* **1,** 211–226.
50. Warheit, D.B., Webb, T.R., Sayes, C.M., Colvin, V.L., and Reed, K.L. (2006) Pulmonary instillation studies with nanoscale TiO2 rods and dots in rats: toxicity is not dependent upon particle size and surface area. *Toxicol. Sci.* **91,** 227–236.
51. Stoeger, T., Reinhard, C., Takenaka, S., Schroeppel, A., Karg, E., Ritter, B., Heyder, J., and Schulz, H. (2006) Instillation of six different ultrafine carbon particles indicates a surface area threshold dose for acute lung inflammation in mice. *Environ. Health Perspect.* **114,** 328–333.

52. Kobayashi, N., Naya, M., Endoh, S., Maru, J., Yamamoto, K., and Nakanishi, J. (2009) Comparative pulmonary toxicity study of nano-TiO(2) particles of different sizes and agglomerations in rats: different short- and long-term post-instillation results. *Toxicology* **264,** 110–118.
53. Grassian, V.H., O'Shaughnessy, P.T., Adamcakova-Dodd, A., Pettibone, J.M., and Thorne, P.S. (2007) Inhalation exposure study of titanium dioxide nanoparticles with a primary particle size of 2 to 5 nm. *Environ. Health Perspect.* **115,** 397–402.
54. Rossi, E.M., Pylkkanen, L., Koivisto, A.J., Vippola, M., Jensen, K.A., Miettinen, M., Sirola, K., Nykasenoja, H., Karisola, P., Stjernvall, T., et al. (2010) Airway exposure to silica-coated TiO2 nanoparticles induces pulmonary neutrophilia in mice. *Toxicol. Sci.* **113,** 422–433.
55. Pan, Z., Lee, W., Slutsky, L., Clark, R.A., Pernodet, N., and Rafailovich, M.H. (2009) Adverse effects of titanium dioxide nanoparticles on human dermal fibroblasts and how to protect cells. *Small* **5,** 511–520.
56. Geiser, M. and Kreyling, W.G. (2010) Deposition and biokinetics of inhaled nanoparticles. *Part. Fibre Toxicol.* **7,** 2.
57. Lu, S., Duffin, R., Poland, C., Daly, P., Murphy, F., Drost, E., MacNee, W., Stone, V., and Donaldson, K. (2009) Efficacy of simple short-term in vitro assays for predicting the potential of metal oxide nanoparticles to cause pulmonary inflammation. *Environ. Health Perspect.* **117,** 241–247.
58. Dick, C.A., Brown, D.M., Donaldson, K., and Stone, V. (2003) The role of free radicals in the toxic and inflammatory effects of four different ultrafine particle types. *Inhal. Toxicol.* **15,** 39–52.
59. Lam, C.W., James, J.T., McCluskey, R., Cowper, S., Balis, J., and Muro-Cacho, C. (2002) Pulmonary toxicity of simulated lunar and Martian dusts in mice: I. Histopathology 7 and 90 days after intratracheal instillation. *Inhal. Toxicol.* **14,** 901–916.
60. Lam, C.W., James, J.T., Latch, J.N., Hamilton, R.F., Jr., and Holian, A. (2002) Pulmonary toxicity of simulated lunar and Martian dusts in mice: II. Biomarkers of acute responses after intratracheal instillation. *Inhal. Toxicol.* **14,** 917–928.
61. Wiessner, J.H., Henderson, J.D., Sohnle, P.G., Mandel, N.S., and Mandel, G.S. (1988) The effect of crystal structure on mouse lung inflammation and fibrosis. *Am. Rev. Respir. Dis.* **138,** 445–450.

### PARTICLE CHARACTERIZATION GLOSSARY

**Acicular:** Elongated particle morphology, where the particle length by far exceeds the diameter, but is still smaller than a fiber (>5 μm long and <3 μm in diameter). This shape may also be denoted a rod.

**Agglomerate[*]:** An assembly of loosely bound particles. The particles are held together by weak noncovalent forces, such as electrical, magnetic, physical entangling, or sticky properties (see Schneider and Jensen[†] for some major mechanisms). The particles can be individualized without breaking a phase. The term coagulation is often used to describe particle agglomeration in air.

**Aggregate[*]:** An assembly of fused or covalently bound particles. The particles are held together by strong chemical bonds or are grown together to form a particle assembly that only can be separated by physically breaking the primary phase(s). In colloid science, the term aggregation is typically used to describe particle agglomeration due to insufficient surface charge or steric stabilization of particles in the dispersions.

**Angular:** The particle contains straight and/or curved (concave/convex) surfaces that do not immediately represent crystalline facets (see euhedral). Crushed materials usually display angular shapes.

**Anhedral:** The particle is rounded and does not display any crystalline faces. A sphere is an example of a fully anhedral particle.

**Euhedral:** The particle displays perfect or near-perfect crystalline faces controlled by the principal atomic structure of the crystalline compound. The particle morphology may also be called idiomorphic.

**Subhedral:** The particle contains both crystalline faces and rounded surfaces, which is not controlled by the principal atomic structure of the compound alone. The particle morphology may also be called subidiomorphic.

**Tabular:** Particle morphology with a table-like form where the lateral extension by far exceeds the horizontal. This morphology may also be called flaky.

[*] It may be difficult to discriminate between agglomerates and aggregates by TEM. It must be emphasized that classification is based on interpretation of the images taken.

[†] Schneider, T. and Jensen, K.A. (2009) Relevance of aerosol dynamics and dustiness for personal exposure to manufactured nanoparticles. *J. Nanopart. Res*. **11(7),** 1637–1650.
